# Excitation of surface plasma waves and fast electron generation in relativistic laser–plasma interaction

**DOI:** 10.1038/s41598-020-70221-9

**Published:** 2020-08-10

**Authors:** M. Raynaud, A. Héron, J.-C. Adam

**Affiliations:** 1 Laboratoire des Solides Irradiés, Ecole Polytechnique, Institut Polytechnique de Paris, CNRS, CEA/DRF/IRAMIS, 91128 Palaiseau, France; 2 Centre de Physique Théorique, CNRS, Ecole Polytechnique, Institut Polytechnique de Paris, 91128 Palaiseau, France

**Keywords:** Physics, Plasma physics, Laser-produced plasmas

## Abstract

The excitation of surface plasma waves (SPW) by an intense short laser pulse is a useful tool to enhance the laser absorption and the electron heating in the target. In this work, the influence of the transverse laser profile and the pulse duration used to excited SPW is investigated from Fluid and 2D Particle-in-Cell simulations. We show the existence of a lobe of surface plasma wave modes. Our results highlight surface plasma waves excitation mechanism and define the laser parameters to optimise the SPW excitation and the kinetic energy of the associated electron trapped in the wave. It opens the door to monitor the spectral mode distribution and temporal shape of the excited surface waves in the high relativistic regime. The most important result of the study is that—at least in 2D—the charge and the energy of the electron bunches depend essentially on the laser energy rather than on temporal or spatial shape of the laser pulse.

## Introduction

The excitation of surface plasma waves (SPW) with laser beam intensity in the range of $10^{18-19}\;\text{ W } \text{ cm}^{-2}\upmu \mathrm{{m}}^2$ is known to be an efficient mean to improve the laser absorption in the target and the generation of fast electrons and ions^1–9^. Of particular interest is the recent experimental demonstration that SPW excitation enhance the creation of highly collimated ten of MeV electron bunches propagating along the plasma surface with 600pC of charge for a laser pulse with an intensity of the order of $2\times 10^{19}\;\text{ W } \text{ cm}^{-2}\upmu \mathrm{{m}}^2$^10^. It is of importance considering the potential applications^11^ in the generation of a bright source of ultrashort pulsed X-rays^12^, ultra-fast electron diffraction, tabletop electron accelerators and ultra-fast electron spectroscopies^13–15^ for example.

The idea of using SPW in over-dense plasma as an alternative approach to enhance the laser absorption and the electron heating has emerged by analogy with the well-known Surface Plasmon (SP) excitation in the solids^16–18^. In this case, the SP is a longitudinal collective oscillation of the electron gas at the solid surface. It is well described by a free electron Drude model which gives for the wave vector mode dispersion $k_{//}=(\omega /c)(\epsilon /(1+\epsilon )^{1/2})$ where $\epsilon$ is the dielectric function of the metal, *c* the speed of light and $\omega$ the frequency of the SP oscillation. SP in solids has been widely investigated in particular with long non-focused laser pulse ($\tau _L\sim 1 ps$ and $I_L\le 10^{10} \;\text{ W } \text{ cm}^{-2}\upmu \mathrm{{m}}^2$) excitation on grating targets^16^. In such situation, it is well known that the SP resonance is very sharp and the experiment conditions for reaching phase matching in case of grating target for example are hard to fulfill. The derivation of this dispersion relation is nevertheless theoretically only valid for gratings very shallow.

When an intense ultrashort ($\tau _L\le 200 \text{ fs }$ and $I_L\sim 10^{18} \;\text{ W } \text{ cm}^{-2}\upmu \mathrm{{m}}^2$) laser pulse is impinging onto a solid target, multiple ionization rapidly occurs without significant ablation. The hydrodynamic expansion over the first cycle of the interaction, partially reduced by the radiative pressure of the laser, has not enough time to smooth the density gradient and an overdense plasma can be created which can keep the steepened density profile of the original target. In such case $\omega _0 \ll \omega _{pe}$, the SPW is in first approximation almost longitudinal and the SP wave dispersion relation can be transposed taking $\epsilon = 1- (\omega /\omega _{pe})^2$ so that we have in the non-relativistic limit (cold-plasma limit and fluid description)^19^: $k_{//}^2 c^2 /\omega ^2 = (1-(\omega /\omega _{pe})^2)/(1-2(\omega /\omega _{pe})^2)$, where $k_{//}$ is the wave vector of the SPW, $\omega _{pe}^2= 4\pi e^2 n_e/m_e$ the plasma frequency, $m_e$ the electron mass and $n_e$ the electron density. However, in plasma experiments the gratings that are used are never shallow. The typical ratio of the modulation depth to the periodicity length is typically 1/2 and no analytic dispersion relation for the SPW even in the non-relativistic limit for such gratings exists. Moreover, in the relativistic regime, no theory at all exists because the electromagnetic wave is non purely transverse in the plasma due to the $\mathbf {J} \times \mathbf {B}$ term. As there is no analytic solution to the Maxwell equations in linear polarisation, writing the field boundary conditions at the plasma–vacuum interface becomes impossible. Although all the limitations mentioned above apply, the non-relativistic cold dispersion relation has been used with success to define the conditions of the SPW excitation with laser beam intensity up to $\sim 10^{18-19}\;\text{ W } \text{ cm}^{-2}\upmu \mathrm{{m}}^2$. It has even been also validated by experimental observations^5^ in the strongly relativistic regime^2^. In the corresponding plasma experiments, the laser has a high contrast (e.g. $\sim 10^{12}$ in Ref.^9^) ultra-short duration and is highly focused, in order to reach high intensity and create a sharp edge over-dense plasma. These conditions are drastically different than those used in the solid case. Experimentally in plasma one finds that a SPW is excited for a relatively large range angle of incidence of the laser of the order $6-7^\circ$^10^ around the theoretical value of the excitation angle of incidence^19^ and a laser contrast greather than $\sim 10^{11}$ is required (see e.g. Ref.^5^). It questions the role of the spectral shape and the temporal duration of the laser in relation with the SPW excitation. This is even more important since with the recent development of intense short pulse laser ($\ge 10^{20}\;\text{ W } \text{ cm}^{-2}\upmu \mathrm{{m}}^2$ and $\le 25\;\text{ fs }$) with very high contrast ($\sim 10^{12}$), SPW in the high relativistic regime can now be explored experimentally to further enhance harmonic generation^20–22^ and the density of the energetic particles produced during the interaction.

To answer these questions, we present in this paper extended Fluid and 2*D* Particle-In-Cell (PIC) simulations of the laser–plasma interaction in conditions of SPW excitation varying the transverse laser profile and the pulse duration extending into the ultra relativistic regime. The structure of the paper is as follows. We first start by describing the numerical settings. The role of the incident transverse laser profile and of the pulse duration on the SPW excited modes is then discussed. The consequence on the electron dynamic is next analysed. Finally we highlight the evolution of the SPW and of the electron dynamic in the highly relativistic regime. The last part of the paper contains discussions and conclusions.

## Results

### Parameters of the simulations

We consider an over-dense plasma with an electron density $n_e=120n_c$ and a steep density gradient along the *x* direction perpendicular to the plasma-vacuum interface in the (*x*, *y*) simulation plane. The plasma is located in the $x>0$ region. Here $n_c =\omega _0^2 m_e/4\pi e^2$ is the critical density with $\omega _0$, the laser frequency. The simulation parameters are chosen as close as possible to Milar target (poly(ethylene terephthalate) –[O–(CH2)2–O–CO–pPh–CO]$_n$–) as in recent experiments^5,9^. The values of Z and A in the simulations are average value taken respectively equals to 4 and 6. The plasma is initially described by a Maxwellian distribution with an initial electron of $T_e=50 \;\text{ eV }$ and an ion temperature of $T_i=T_e/5$. The choice of this temperature is discussed in the section “Methods”.

In order to fulfill the matching condition of the surface wave excitation, we take a modulated sinusoidal surface with a modulation periodicity d ($\hbox {d}=8.44 k_0^{-1}\sim 1\;\upmu \mathrm{{m}}$) along the *y*-direction. The SPW wave is excited by coupling with one of the electromagnetic waves diffracted by the grating whose wave numbers are defined by $k_y=k_0sin\theta _i + 2\pi n/d$ where $\theta _i$ is the laser angle of incidence, $k_0$ the laser wave vector and *n* an integer. Using the non-relativistic cold dispersion relation for the chosen plasma density one find for $\omega =\omega _0$ that the wave vector of the SPW is $k_y=k_{//}=1.0036 k_0$.With the chosen grating periodicity, the SPW is excited at an incidence angle of the laser of $15^\circ$ by the first order diffracted mode. The dip-to-tip modulation depth h of the modulated surface equals $2.094 k_0^{-1}$ that is h/d$\sim 0.25$.

The incoming oblique laser beam of intensity ranging from $2.6\times 10^{19}\;\text{ W } \text{ cm}^{-2}\upmu \mathrm{{m}}^2$ (which corresponds to the dimensionless parameter $a_0=eE/{m_e\omega _0c}=4.4$) to $1.3\times 10^{21}\;\text{ W } \text{ cm}^{-2}\upmu \mathrm{{m}}^2$ ($a_0=31$) is *P*-polarized (magnetic field perpendicular to the (*x*, *y*) simulation plane). The time dependence of the laser field exhibits a truncated Gaussian shape corresponding to a pulse duration of $\tau _L=70 \omega _0^{-1}$ (FWHM $\simeq 20 \;\text{ fs }$ with $\lambda _0=0.8\;\upmu \mathrm{{m}}$ the laser wavelength). For spatial dependence, we adopted a Gaussian transverse profile with a width, $Y_{p_w}$, ranging from $30k_0^{-1}$ ($\sim 3.8\;\upmu \mathrm{{m}}$) to $240k_0^{-1}$ ($\sim 30.4\;\upmu \mathrm{{m}}$) that is focused onto the center of the plasma surface at $k_{0}x = k_{0}y=0$. In the presented simulations, the maximum impulsion of the incident laser is impinging on the plasma surface at the time $\omega _0 t=0$.

The size of the Fluid simulation box is: $2136k_0^{-1}$ along the *y*-direction ($340\lambda _0\sim 272\;\upmu \mathrm{{m}}$) and $250k_0^{-1}$ along the *x*-direction ($38.2\lambda _0\sim 31.6\;\upmu \mathrm{{m}}$) in front of the plasma. In order to keep the same description as the fluid one, we take for the Particle-In-Cell (PIC) simulations the same transverse size of the simulation box. Along the perpendicular *x*-direction at the front of the plasma we use a larger simulation box size ($103\lambda _0\sim 82.4\;\upmu \mathrm{{m}}$) and the same dimension at the rear of the plasma to avoid that the fastest particles reach the boundaries too rapidly. In all the figures, the units are $k_0^{-1}$ for space, $\omega _0^{-1}$ for time, $n_c$ for density and $m_e\omega _0/e$ for magnetic field.

### Role of the incident transverse laser profile on the SPW excited mode

**Figure 1:**
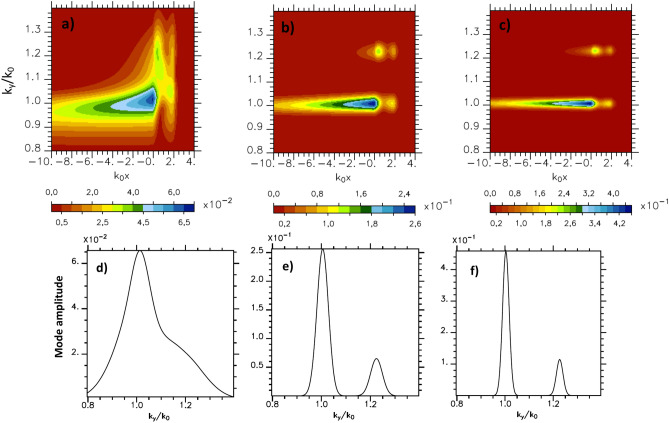
$B_z$ component of the magnetic fields for $a_0=4.4$ and $\tau _L=70 \omega _0^{-1}$ in the Fourier space ($k_0x$, $k_y/k_0$) on the vacuum side in front of the grating (at $\omega _0 t=0$, the plasma is located in the $x>0$ region): (**a**) $Y_{p_w}=30k_0^{-1}$, (**b**) $Y_{p_w}=120k_0^{-1}$ and (**c**) $Y_{p_w}=240k_0^{-1}$. Spectral mode distribution of $B_z$ component of the magnetic fields (at $k_0x=-0.2$): (**d**) $Y_{p_w}=30k_0^{-1}$, (**e**) $Y_{p_w}=120k_0^{-1}$ and (**f**) $Y_{p_w}=240k_0^{-1}$.

To investigate the role of the spatial Gaussian transverse profile width $Y_{p_w}$ of the incident laser field, we first perform a set of fluid simulations increasing $Y_{p_w}$ from $30k_0^{-1}$ up to $240k_0^{-1}$ keeping unchanged the other parameters. So doing we study the influence of the laser focalization varying the local laser incidence distribution around the mean incidence (here $15^\circ$). As $\omega _0 \ll \omega _{pe}$ and the phase velocity $v_\varphi = \omega /k_y$ of the SPW is close to *c*, the SPW field components are such that $B_z \sim E_x \gg E_y$. Therefore we will in the following discuss the SPW field evolution thought the analysis of the $B_z$ component. In order to characterize the surface plasma wave, we evaluate the Fourier transform in the y direction of the $B_z$ component of the magnetic field at any point x of the simulation box, this Fourier transform being performed over the whole simulation box size in y direction . We show in Fig. 1a–c the dependence of the $B_z$ field as a function of $k_0x$ and $k_y / k_0$ for the different focal spots. We observe a lobe of maximum amplitude around $k_y / k_0 = 1$ corresponding to the mode n = 1 diffracted by grating, extending in front of the plasma and another mode of weaker amplitude located at the plasma interface at $k_y/k_0=1.23$ which corresponds in the mode ($\hbox {n} = -2$) diffracted by grating.

For our physical parameters, the mode ($k_y/k_0=1.0036$) corresponds to SPW. This mode has the highest amplitude on the plasma surface but we do not observe a sharp resonance due to the short duration of the laser pulse^23^. We also observe an exponential spatial decrease of its amplitude in the direction perpendicular to the plasma surface. This decrease is proportionate to $e^{x/L_v}$ with $L_v$ the length of the decrease equals to $\sim 7.7 k_0^{-1}$ in rather good agreement with the theoretical value $9.2 k_0^{-1}$ obtained considering a mono-mode situation^8^. The Fig. 1d–f are the cuts of the Fig. 1a–c at $k_0x=-0.2$. We notice that the width of the spectral mode distribution decreases when increasing the Gaussian transverse profile width $Y_{p_w}$ of the incident laser field. So, in case of an infinite transverse profile, a mono-mode situation would be reached. The reduction of the spectral mode distribution is accompanied by an increase of the mode amplitude that matches the increase of the corresponding spectral component of the incident beam. It can be attributed to an increase of the incident laser energy. We checked by comparing a simulation with $Y_{p_w}=30k_0^{-1}$ ($a_0=4.4$) and one (not reported here) with $Y_{p_w}=240k_0^{-1}$ ($a_0=0.5475$) corresponding to the same incident laser energy (keeping all the other parameters unchanged) that there was no variation of the mode amplitude of the SPW. As expected, the spectral width of the SPW only depends on the width of the laser pulse.

**Figure 2:**
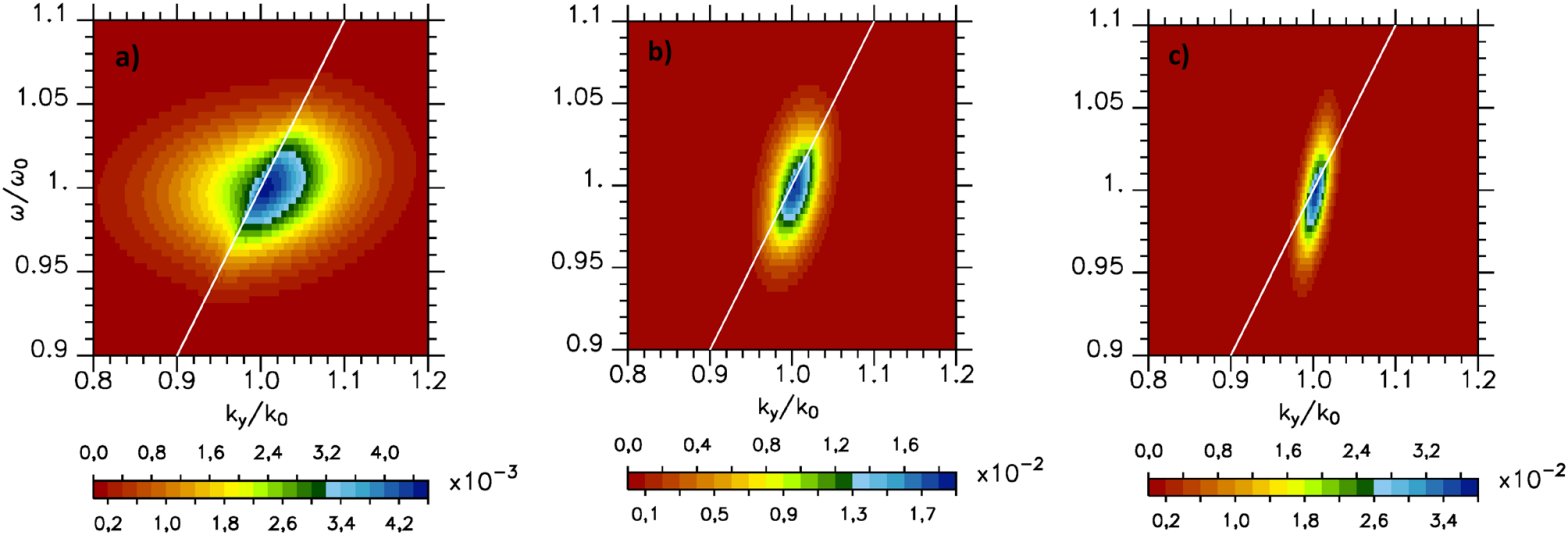
Spectral mode distribution in the $(k_y/k_0,\omega /\omega _0)$ space of $B_z$ component of the magnetic fields for $a_0=4.4$ and $\tau _L=70 \omega _0^{-1}$ (at $k_0x=-0.1$): (**a**) $Y_{p_w}=30k_0^{-1}$, (**b**) $Y_{p_w}=120k_0^{-1}$ and (**c**) $Y_{p_w}=240k_0^{-1}$. A white straight line showing $\omega /k_0c=1$ is added. It separates on the left side: propagating modes in the *x* and *y*-direction, and on the right: SPW modes that are evanescent in the *x*-direction and propagating in the y one.

**Figure 3:**
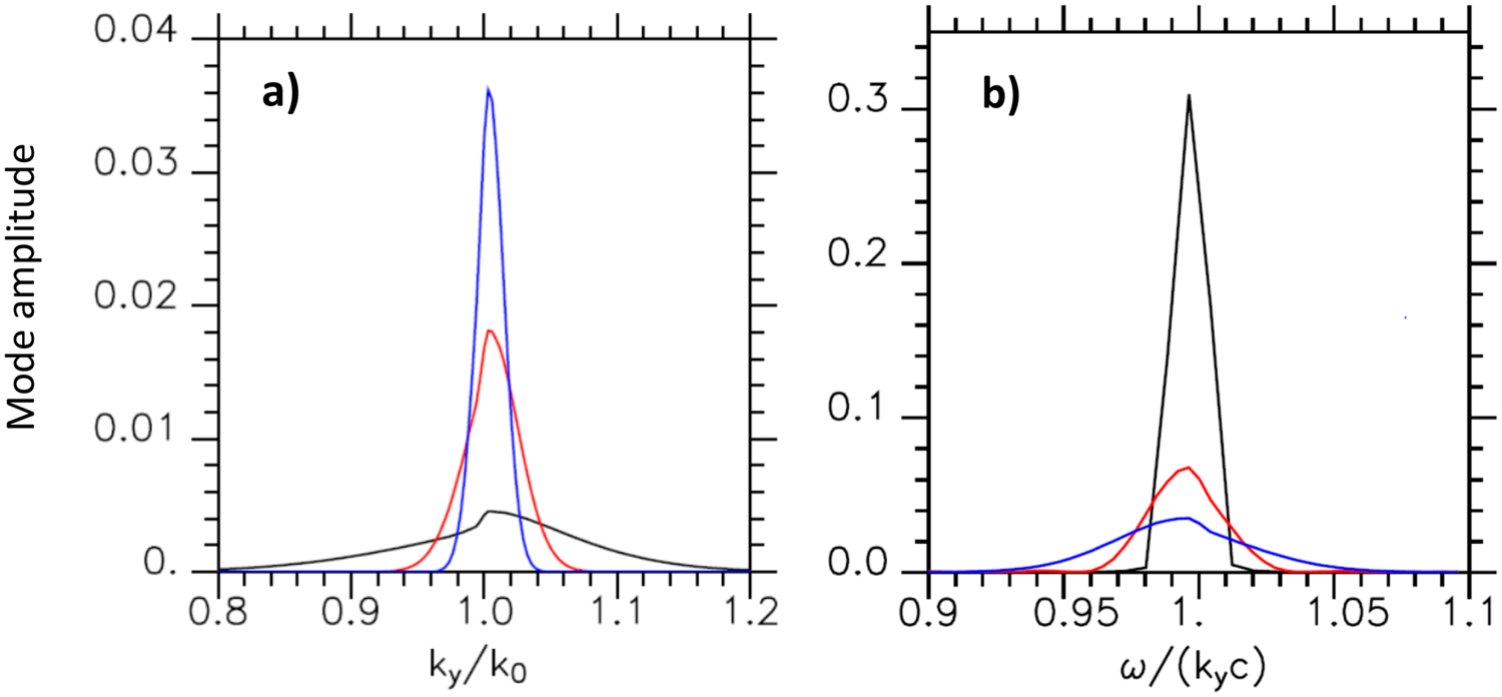
(**a**) Modes distribution as a function of $k_y/k_0$ for $\omega =\omega _0$ in case $\tau _L=70 \omega _0^{-1}$ and for $Y_{p_w}=30k_0^{-1}$ (in black), $120k_0^{-1}$ (in red) and $240k_0^{-1}$ (in blue); (**b**) modes distribution as a function of $\omega /k_yc$ for $k_y=1.0033k_0$ and $Y_{p_w}=240k_0^{-1}$ in case $\tau _L=70 \omega _0^{-1}$ (in blue), $140 \omega _0^{-1}$ (in red) and $\infty$ (in black). (**a**) is obtained as a cut of Fig. 2.

For a given spatial value *x*, we calculate the Fourier transform in time of the field and plot the mode distribution in the $(k_y,\omega )$ space (see Fig. 2). On this figure we also add a white straight line showing $\omega /kc=1$. It separates on the left side propagating modes in the *x* and *y*-direction, and on the right, modes corresponding to SPW that are evanescent in the *x*-direction and propagating in the y one. Figure 2 shows that the part of the lobe corresponding to SPW concentrates the larger part of the energy. We note that the width in frequency of the SPW is the same as that of the incident laser beam which is kept constant in this set of simulations. Moreover it does not vary increasing the Gaussian transverse profile width of the incident laser field. Although the maximum amplitude of the mode lobe is at $\omega =\omega _0$ and $k_y=1.0033k_0$, its extension in *k*-space becomes larger as the focusing of the incident exciting beam increases. This is even more visible in Fig. 3a where we have drawn as a cut of Fig. 2 the mode distribution as a function of $k_y/k_0$ for $\omega =\omega _0$ for three different spectral width. In the limit of a pulse of infinite width (although this situation is somewhat unrealistic from an experimental point of view), only one mode would be excited in agreement with the SPW model of Kaw^19^.

Hence the use in experiments of very short focused laser beam ($\le 25\;\text{ fs }$ and waist $\le 4\;\upmu \mathrm{{m}}$) allows large spectral modes excitation whereas with lesser focused laser beam mono mode excitation with higher intensity is obtained and the matching condition of the surface wave excitation is more difficult to fulfill. It explains why SPW excitation using highly focused incident laser beam with high contrast to prevent plasma expansion at the beginning of the interaction is easier than SP excitation in solid with unfocused laser.

### Role of the incident laser pulse duration on the SPW excited mode

We consider in this section fluid simulations in which we increase the pulse duration and kept unchange the Gaussian transverse profile width of the incident laser field. We analyze the mode evolution drawing the mode distribution as a function of $\omega /k_yc$ for $k_y/k_0=1.0033$ (see Fig. 3b). Increasing the pulse duration from $\tau _L=70 \omega _0^{-1}$ ($20\text{ fs }$) to $140 \omega _0^{-1}$ ($40\text{ fs }$) while keeping all the other parameters constant decreases the spectral width in frequency by a factor 2. The mode amplitude follows the laser energy increase. It should be emphasized here that the lobe of SPW excited modes extends up to $\omega /k_yc > 1$ which is of particular importance for experiments in which high electron acceleration is sought. We will study in the following the impact of the SPW on the electron acceleration along the plasma surface.

### Laser focusing and electron acceleration

**Figure 4:**
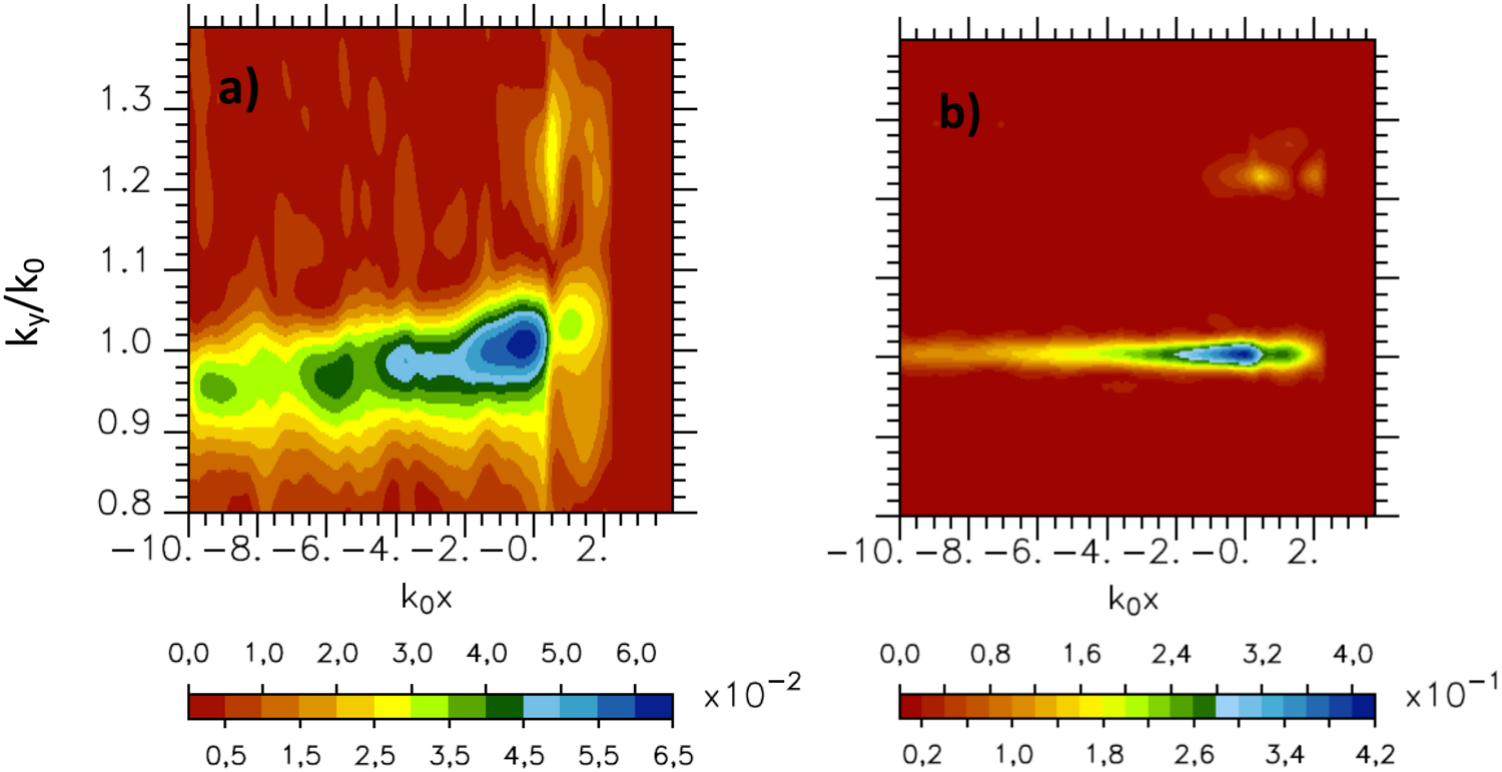
PIC simulations: spectral mode distribution in the Fourier space ($k_0x$, $k_y/k_0$) of $B_z$ component of the magnetic fields for $a_0=4.4$ (at $\omega _0 t=0$) on the vacuum side in front of the grating (the plasma is located in the $x>0$ region) for: (**a**) $Y_{p_w}=30k_0^{-1}$ and (**b**) $Y_{p_w}=240k_0^{-1}$.

To study the electron dynamic, the Fluid simulations have been complemented by two-dimensional Particle-In-Cell (PIC) calculations with the relativistic collisional code EMI2D^24^. Comparison of the fields obtained with PIC simulations and fluid ones shows that they are very similar in shape as seen in Fig. 4 where we report the Fourier transform of the $B_z$ field in the case $Y_{p_w}=30k_0^{-1}$ and $240k_0^{-1}$ for $a_0=4.4$ in PIC simulations. As previously seen, we note that the width of the spectral mode distribution decreases when increasing the Gaussian transverse profile width of the incident laser field up to $Y_{p_w}=240k_0^{-1}$ (see Fig. 4b). It confirms how powerful the fluid approach to make parametric studies is. However it should be emphasized that the field amplitude in the fluid simulations depends on the collisional term value $\nu _{ei}/\omega _0$ , which is somewhat arbitrary given its absence of evolution as a function of plasma temperature in the present model.

**Figure 5:**
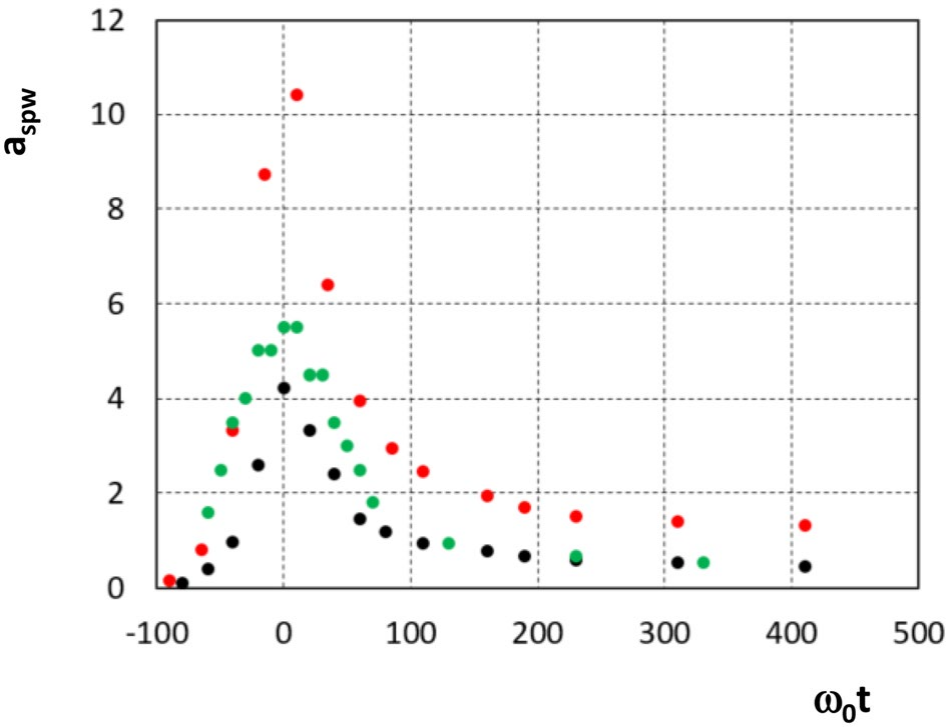
Rebuilded SPW field amplitude $a_{SPW}= eE_{SPW}/m_ec\omega$ obtained from the inverse Fourier transform of the modes such that $0.8\le k_y/k_0\le 1.2$ ($\tau _L=70 \omega _0^{-1}$): case $Y_{p_w}=30k_0^{-1}$ and $a_0=4.4$ (black dot), case $Y_{p_w}=240k_0^{-1}$ and $a_0=4.4$ (green dot) and case $Y_{p_w}=30k_0^{-1}$ and $a_0=13.7$ (red dot).

During the high-intensity short-pulse interaction between the laser and the over-dense plasma, relativistic electrons are produced with non-thermal distribution^25^ which can be used to generate energetic ions at the rear surface of the target^26^. The most efficient mechanisms are here the so-called ponderomotive or $\mathbf {J} \times \mathbf {B}$ heating^27,28^ and vacuum heating^29,30^. In this context, SPW excitation can be used to increase the fast electromagnetic-energy transfer to the plasma due to electron heating by collisionless mechanisms. When the SPW field is large enough, electrons from the plasma surface that are initially at rest can be injected with high efficiency into the SPW, due to the $\mathbf {v} \times \mathbf {B}$ force. If the direction of propagation of the electrons coincides with that of the SPW, they can be phase-locked and trapped in the wave^8^. One can then expect that the acceleration will be strong enough to create a bunch of electrons travelling along the plasma/vacuum interface with relativistic velocities as the phase velocity $v_\varphi = \omega /k_y$ of the SPW is according to the value of the SPW wave vector^19^ close to *c* ($v_\varphi /c = \omega /k_y \sim 0.996$ taking from the non-relativistic cold dispersion of the SPW $k_y=k_{//}=1.0036 k_0$). The model described in Ref.^8^ evidences the role in the relativistic regime of 2D effects which enhance the maximum electron kinetic energy, $E_{kin}$, of the electrons trapped into the SPW. These electrons travel along the surface with a kinetic energy $E_{kin}\sim \gamma _\varphi a_{SPW} m_e c^2$ with $a_{SPW}= eE_{SPW}/m_ec\omega$. Here $E_{SPW}$ is the maximum field amplitude of the SPW at the time of electron injection and $\gamma _\varphi =(1-(v_\varphi /c)^2)^{-1/2}$. We notice that this model over-estimates $E_{kin}$ by neglecting any further SPW field relaxation during the propagation of the electron bunch.

To compare our PIC simulations with this model, we first determine the maximum SPW field amplitude, $a_{SPW}$, extracted from the rebuilded SPW field obtained from the inverse Fourier transform of the modes such that $0.8\le k_y/k_0\le 1.2$. The field amplitude evolution with time is reported in Fig. 5 in black in the case $\tau _L=70 \omega _0^{-1}$, $Y_{pw}=30k_0^{-1}$ and $a_0=4.4$. We observe an abrupt raise of the field amplitude when the laser field impinges the plasma surface followed by a quick damping over $\sim 60\omega _0^{-1}$. In order to test the role of the collisional damping, we also compare two simulations with the same parameters suppressing in the last one the collisional term (not reported here). As we notice no change in the SPW field which propagates on the surface of the plasma, we attribute the quick SPW damping to energy transfer to the electrons. In the case $Y_{p_w}=30k_0^{-1}$, we found $a_{SPW}=4.22=0.96a_0$ so that the maximum kinetic energy of the electron expected with this model is $\sim 24\;\text{ MeV }$.

**Figure 6:**
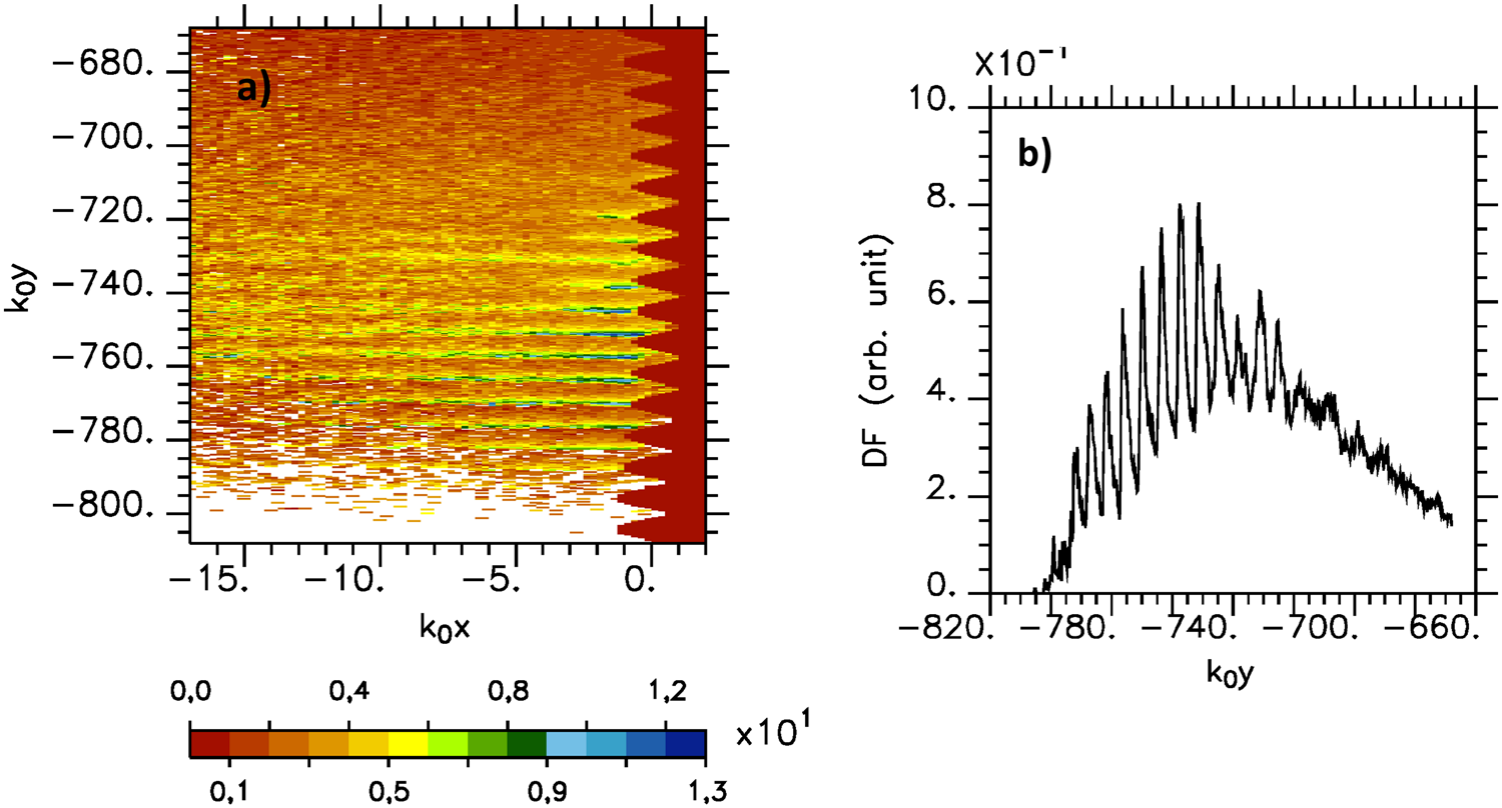
Kinetic energy in $\text{ MeV }$ (**a**) and distribution function, DF, (**b**) of the kinetic energy of the electron bunches which propagate on the plasma surface average over $-17 \le k_0x \le 0$ at the end of the simulation ($\omega _0 t=750$, $a_0=4.4$, $Y_{p_w}=30k_0^{-1}$).

We now concentrate on the electron population at the plasma/vacuum interface of the simulation. Considering the plot of the electron kinetic energy drawn in Fig. 6, we observe electron bunches highly located near to the grating surface ($-17<k_0 x<0$) and propagating along the plasma/vacuum interface. The most energetic ones are confined in the parallel direction on roughly $2Y_{p_w}$. The maximum energy reached is about $12.9\;\text{ MeV }$ and is half of the maximum energy predicted by the model^8^. This result points out its limitation as it deals with an ideal situation where electrons are injected and trapped in a mono mode SPW situation. We also observe multi pics in the distribution function of the kinetic energy at periodicity that does not depend on the grating parameter. Theses pics are the macroscopic signature of phase bunching effects predicted in the model^8^ and corresponding to those electrons that have seen the most favorable phase of the SPW field during the whole interaction time. We notice that this multi peaked kinetic energy distribution is observed soon at the very beginning of the SPW excitation and survives during all the simulation time. It is not observed if the surface plasma is plane or if the grating parameter does not satisfy the condition for SPW excitation.

Increasing the Gaussian transverse profile width of the incident laser beam $Y_{p_w}$ from $30k_0^{-1}$ up to $240k_0^{-1}$ (keeping unchanged the other parameters) enhances the overall spatial extension of the electron distribution which propagates along the plasma surface. We also note that this increases the laser energy by a factor 8 in 2D geometry. As expected no change in the periodicity of the bunches is observed but an increase of the maximum electron kinetic energy is found which reaches $25.8\;\text{ MeV }$. This increase of a factor 2 is much higher than one of the maximum SPW field amplitude which is only $30\%$ larger (see Fig. 5 green points) and corresponds to $a_{SPW}=5.5$. It is related to the fact that increasing the Gaussian transverse profile width of the incident laser beam also enhances the length of interaction. This result is in agreement with the result of Ref.^8^.

**Figure 7:**
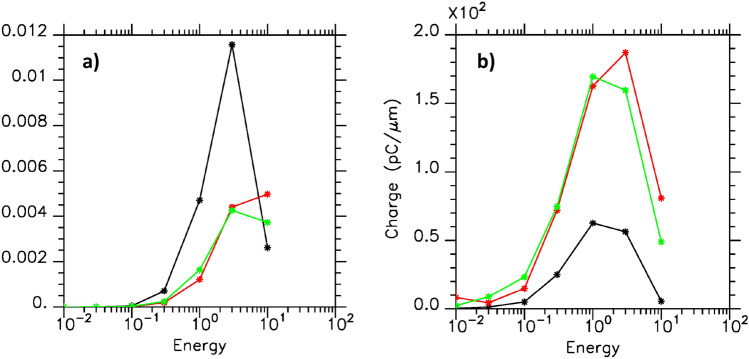
(**a**) Fraction of the laser energy (in %) converted in electron as a function of the electron kinetic energy (in *MeV*); (**b**) Charge (${\mathrm{pCm}}^{{ - 1}}$). The electrons considered are those in the space ($-17 \le k_0x \le 0$, $-820 \le k_0y \le -660$) at the end of the simulation ($\omega _0 t=750$). Black stars: case $a_0=4.4$, $Y_{p_w}=30k_0^{-1}$; green stars: case $a_0=4.4$, $Y_{p_w}=240k_0^{-1}$; red stars: case $a_0=13.7$, $Y_{p_w}=30k_0^{-1}$.

To complete this analysis, we estimated from the simulations the fraction of the laser energy that was converted into kinetic energy of the electron beam propagating along the plasma surface with the SPW mode as a function of the electron kinetic energy. This has been done using the code diagnostic of the spectrum of the electron energy density analyzed according to a logarithmic scale. Integrating this diagnostic over space (limited in the transverse *x*-direction from $-17 k_0^{-1}$ to the plasma surface, width which allows taking into account all the energetic electrons travelling along the plasma surface in the SPW field), one gets the average distribution of electrons. Figure 7a represents this distribution divided by the total laser energy injected in the system as a function of the electron energy in *MeV*. It gives the fraction of laser energy converted into fast electrons (travelling along the surface) of energy between *E* and $\sqrt{10}E$ as a function of *E*. For $E=10\;\text{ MeV }$ all the electrons with higher energy than $10\;MeV$ are taken into account. In Fig. 7b, we have reported the corresponding charge expressed in ${\mathrm{pC\mu m}}^{{ - 1}}$.

We observe in Fig. 7a a fraction of the converted laser energy smaller as the width of the incident laser beam $Y_{p_w}$ increases from $30k_0^{-1}$ (in back) up to $240k_0^{-1}$ (in green) (keeping unchanged the other parameters). We notice however that the increase of $Y_{p_w}$ corresponds to a laser energy eight time greater which explains the increase of the charge observed in Fig. 7b.

### Very high relativistic regime

In experiments^31–34^, increasing laser intensity in the high relativistic regime is mostly obtained with highly focused ultra-short duration pulse. As just discussed, this has consequences on the SPW mode distribution. Moreover, laser intensities larger than $10^{18}\;\text{ W } \text{ cm}^{-2}\upmu \mathrm{{m}}^2$ induce relativistic detuning already observed in fluid simulations^23^. As a consequence of the reduction of the effective plasma density, the k-wave vector of the SPW will be shift to higher value which will change the phase matching conditions.

**Figure 8:**
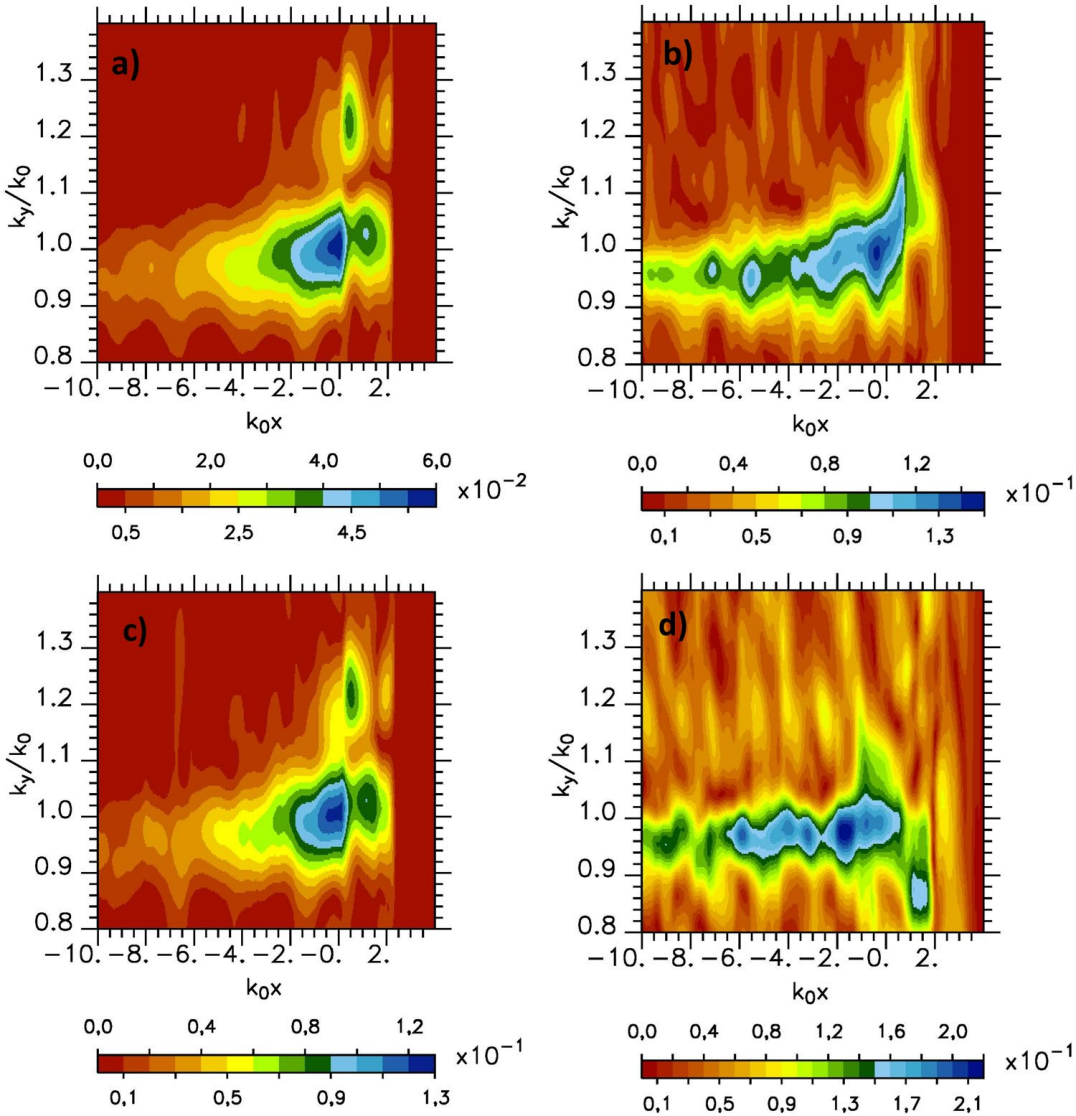
PIC simulations: spectral mode distribution in the Fourier space ($k_0x$, $k_y/k_0$) of $B_z$ component of the magnetic fields for $Y_{p_w}=30k_0^{-1}$ (**a**,**c**) at $\omega _0 t=-50$ and (**b**,**d**) at $\omega _0 t=0$) on the vacuum side in front of the grating (the plasma is located in the $x>0$ region) for (**a**,**b**) $a_0=13.7$ and (**c**,**d**) $a_0=31$.

In the following in order to investigate the SPW in these conditions, we have performed 2*D*-PIC simulations increasing the laser intensity from $a_0=4.4$–31 (keeping all the other parameters unchanged). Up to this value of $a_0$ we continue to observe SPW excitation although the field structure around $k_y/k_0\sim 1$ becomes disturbed as seen on Fig. 8 where we report for $a_0=13.7$ and $a_0=31$ the spectral mode distribution in the Fourier space ($k_0x$, $k_y/k_0$) of $B_z$ component of the magnetic fields at two time $\omega _0 t=-50$ on the left and $\omega _0 t=0$ on the right on the vacuum side in front of the grating. At the beginning of the interaction ($\omega _0 t=-50$) the mode structure in the Fig. 8a,c is close to one observe in the low intensity case (see to compare the Fig. 4). Increasing the time of interaction, the mode structure in the Fig. 8b,d is altered especially as the value of $a_0$ increases. This is due to the increase of the laser pressure which quickly deforms the plasma surface in this high intensity regime. In return it modifies the field shape. This deformation is particularly large as we simulated a Milar target which yields an effective relativistic density of approximately 4 for $a_0=31$. The expansion of the ion density also contributes to perturb the SPW field structure. Nevertheless, the SPW excitation appears to be robust during the first cycles of the interaction despite the relativistic detuning of the mode distribution^23^. The average spatial extension of the SPW field in the direction perpendicular to the plasma surface remains exponential as in the weak relativistic regime and fluid simulations. The rebuilded SPW field amplitude obtained from the inverse Fourier transform of the modes such that $0.8\le k_y/k_0\le 1.2$ in case $a_0=13.7$ gives $a_{SPW}=0.76a_0$ (see Fig. 5). In case $a_0=31$ the SPW field structure is too complex due to surface plasma profile deformation to extract a valuable value of $a_{SPW}$. Nevertheless the SPW field deformation in the high relativistic regime does not prevent further electron acceleration along the surface of the plasma. The maximum electron kinetic energy obtained in the simulations for electrons travelling along the plasma surface is $23.6\;\text{ MeV }$ in the case $a_0=13.7$ and $43.5\;\text{ MeV }$ in the case $a_0=31$. The simulation for $a_0 = 31$ was followed over a sufficiently long time to allow us to highlight the existence of the surface wave in the ultra relativistic regime but too short to study the characteristics of the electron beam propagating along the plasma.

We notice that increasing the laser intensity from $a_0=4.4$ to 13.7 (keeping unchanged the other parameters) increases the laser energy by a factor $\sim 10$. The increase of the width of the incident laser beam in case $a_0=4.4$ from $Y_{p_w}=30k_0^{-1}$ to $240k_0^{-1}$ also increases the laser energy by a factor $\sim 8$. Thus when looking to the fraction of the laser energy converted in electron kinetic energy and to the charge, we found a similar behavior between the case $a_0=4.4$ with $Y_{p_w}=240k_0^{-1}$ (Fig. 7a,b in green) and the case $a_0=13.7$ with $Y_{p_w}=30k_0^{-1}$ (Fig. 7a,b in red). It appears here that in these two dimensional simulations, the energy transfer and the charge of the electron bunches seem more dependent on the incident laser energy than on the structure of its focal spot.

Finally we report in the Table 1 the value of the absorption of the laser beam in the plasma target (electrons and ions) and different characteristics of the bunch of electrons propagating along the plasma surface. We give the maximum kinetic energy of these electrons and adding the results of Fig. 7 for the electrons with an energy higher than $3\;\text{ MeV }$ we obtained the fraction of the laser energy converted into this electron beam and the corresponding charge expressed in ${\mathrm{pC\mu m}}^{{ - 1}}$. We observe a high laser absorption in this short pulse laser grating interaction scheme which induces SPW excitation. We notice that increasing the laser intensity do not reduce this high laser absorption. When the transverse laser profile is increased keeping the laser intensity constant, we also note an increase in absorption. We attribute this behavior to a geometrical effect: increasing the surface of the focal spot enhances the surface of the laser plasma interaction and subsequently the generated charge. In order to compare with previous experimental measurements, we estimate the charge in pC of the electron bunch in 3D geometry by integrating the charge per micrometer on half of the focal spot in a fake direction *z*. The values obtained are given in the Table 1. We should outline here that this estimation is obtained from 2*D* simulations.

**Table 1:** Absorption, maximal electron kinetic energy in *MeV*, fraction of the laser energy (in electron kinetic energy, charge in ${\mathrm{pC\mu m}}^{{ - 1}}$ and charge in pC of the electron bunch obtained by integration on half of the focal spot in a fake direction *z*.

$a_0$	$Y_{p_w}$	Absorption (%)	Maximal electron kinetic energy (*MeV*) given to the electrons	Fraction of energy (%)	Charge (${\mathrm{pC\mu m}}^{{ - 1}}$)	Estimated charge on half of the focal spot (pC)
4.4	30$k_0^{-1}$	53	12.9	1.4	62	118
4.4	240$k_0^{-1}$	69	25.8	0.94	208	3167
13.7	30$k_0^{-1}$	54	23.6	0.8	268	509

## Discussion

The results obtained in this paper shed new light on the nature of the SPW in over-dense plasma. We evidence a lobe of SPW modes with phase velocity extending up to *c* that allows acceleration of electrons along the wave direction of propagation i.e. the plasma surface. The electron acceleration can be related to the SPW field amplitude which propagates along the plasma surface and to the duration of the interaction. The maximum electron kinetic energies found in our PIC simulations are in agreement with the model prediction reported in Ref.^8^. This study also highlights for future experiments the importance of laser parameters such as pulse duration and spectral width for surface plasma wave excitation using various surface structuring. The understanding of the excitation mechanism of the SPW in the high relativistic regime given in this paper opens new possibilities to monitor its spectral mode distribution and temporal shape which is of particular importance for further optimization of intense ultra-short electron sources in the $\;\text{ MeV }$ range. The 2D results suggest that an increase of the laser energy, either by an increase of the focal spot for a given laser intensity or by an increase of the laser intensity for a given focal spot, yields an increase in the density of the bunch of electrons as well as of its energy. Nevertheless it is important to realize that these dependencies should be confirmed by 3D simulations because the laser energy deposited depends on the size of the focal spot in $Y_{p_w}^2$ rather than $Y_{p_w}$ in 2D. Moreover, if increasing the laser intensity will act positively on the density of the bunch of electrons as well as of its energy, SPW excitation is conditioned by strong laser contrast^5^ greather than $\sim 10^{11}$ as prepulse inducing surface plasma expansion will prevent the excitation of the waves, a limiting point of the approach.

## Methods

The fluid model of plasma used is strictly identical to the Drude model of solid-state physics. We consider a grating made of a plasma of electrons neutralized by a fixed background of ions. The presence of a collisional dissipation is described in the model by a collisional term $\nu _{ei}/\omega _0=5$. We consider the relativistic equation of momentum conservation which gives the evolution equation for current used as a source term in Maxwell equation: $\partial j_e/\partial t =-(n_e e)/(m_e \gamma ) (E+v_e\times B)-\nu _{ei} j_e$ where $\gamma$ is the relativistic factor. The code used to integrate Maxwell equations is the same one as the one used next in the PIC code developed at CPhT Ecole Polytechnique^24^, the only difference being the way the current is obtained. We use this approach that has been shown in Ref.^23^ to be useful to perform parametric studies to optimize the conditions of interaction with gratings.

The fluid simulations are complemented by an extended set of 2D Particle-In-Cell (PIC) simulations which allows the electron dynamics description. The PIC simulations are performed in case of mobile ions with the relativistic collisional PIC code EMI2D^24^ The temperature was chosen rather arbitrarily small enough for the collisional effects to play a role but not too small to avoid having a spatial mesh too small to handle it. We have also studied the influence of the initial plasma state: in the first case, we suppressed the collisions keeping the initial temperature of 50 eV, and in a second one, we run a simulation without collisions and an initial temperature of 1 keV. The absorption is identical to the initial collisional model, the various curves being undistinguishable. This is due to the fact that the interaction is localized in a thickness comparable to the skin depth, which heats very rapidly. In order to keep the same description as the fluid one, we take the same transverse size of the simulation box. Along the perpendicular *x*-direction at the front of the plasma we use a larger simulation box size to avoid that the fastest particles reach the boundaries too rapidly. The size of the simulation in the *x*-direction in the front of the plasma is also chosen large enough to have the complete incident laser beam before the plasma interaction so that an exact calculation of the incident laser energy can be made.

The EMI2D code allows using mesh sizes of up to $20\lambda _D$ without self-heating (where $\lambda _D=v_{th}/\omega _{pe}$ is the Debye length). Thus we choose a grid size of $k_0\bigtriangleup x=k_0\bigtriangleup y=0.018\approx 20k_0\lambda _D$ and the number of particles per cell was 120. The mesh size and the dimensions of the simulation box prohibit 3*D* simulations.
